# Inadequate Regulation Contributes to Mislabeled Online Cannabidiol Products

**DOI:** 10.15844/pedneurbriefs-32-3

**Published:** 2018-06-18

**Authors:** Daniel A. Freedman, Anup D. Patel

**Affiliations:** 1 Departments of Pediatrics and Neurology, Nationwide Children’s Hospital, Columbus, OH

**Keywords:** Cannabidiol, Medical Marijuana, Pediatric Neurology

## Abstract

Researchers from the University of Pennsylvania, Veterans Affairs San Diego, RTI international, Americans for Safe Access, Palo Alto University and Johns Hopkins University analyzed the content of 84 cannabidiol (CBD) products purchased on the internet and compared the results to their advertised concentrations.

Researchers from the University of Pennsylvania, Veterans Affairs San Diego, RTI international, Americans for Safe Access, Palo Alto University and Johns Hopkins University analyzed the content of 84 cannabidiol (CBD) products purchased on the internet and compared the results to their advertised concentrations. Products were mislabeled with 26% containing less CBD than labeled and 43% containing more, indicating a high degree of variability and poor standardization of online products. Notably, the oil-based products were more likely to be accurate (45% compared to 25% for tincture and 12.5% for vaporization liquid) and had a smaller percentage of deviation. Oil based products also had a higher range of concentration. In addition to CBD mislabeling, Δ-9-tetrahydrocannabibolic acid (THC) was detected in 21% of samples. This study also notes that products containing THC could have sufficient enough concentrations to produce intoxication in children. [1]

COMMENTARY. The results of this study suggest that despite an FDA warning letter in 2016, the online marketplace for CBD products continues to lack standardization and has a high rate of mislabeling [1]. There are more than 60 cannabinoids in the marijuana plant *Cannabis sativa*. The psychoactive cannabinoid that leads to recreational use of marijuana is THC, which activates the CB_1_ and CB_2_ receptors. CBD does not activate these receptors and does not have psychoactive properties [2]. Emerging evidence suggests that CBD may be useful in the treatment of certain epilepsy syndromes [3,4]. Limited data is available for other neurological diseases(2). Neurologists should be aware of the potential for drug-drug interactions between CBD products and other medications metabolized by CYP2B isozymes, such as clobazam and valproate [5]. Recent evidence suggests that CBD can also significantly change serum levels of rufinamide, topiramate, zonisamide and eslicarbazepine. Variable concentrations of CBD products could have significant impact on serum levels of other antiepileptics and increase liver enzyme levels in patients on valproate [5]. Furthermore, this study should raise concern regarding the percentage of samples with detectable THC levels (21%). The potential long-term effects of recreational marijuana high in THC on the pediatric brain include changes in brain volume and correlation with overall lower cognitive functioning [6]. Although there are no reports to suggest high CBD content products have similar effects, studies are ongoing to investigate for long term risk associations [7].

Overall, the results of this study are an important contribution to the growing evidence that online CBD products have a high rate of mislabeling. A need exists for consistency and regulation of these products. There is potential for adverse events by having higher CBD concentrations than expected, resulting in changing serum antiepileptic levels. Given the variable concentrations of CBD and concern for THC content, these products should be avoided by pediatric patients.

## Disclosures

The author(s) have declared that no competing interests exist.
